# Immunomodulation of Skin Cancer

**DOI:** 10.3390/ijms241310462

**Published:** 2023-06-21

**Authors:** Nabiha Yusuf

**Affiliations:** Department of Dermatology, University of Alabama at Birmingham, Birmingham, AL 35294, USA; nabihayusuf@uabmc.edu; Tel.: +1-205-934-7432; Fax: +1-205-934-0532

Skin cancer represents a major public health issue with a tremendous cost to healthcare systems in the United States and worldwide [1]. Epidemiological studies indicate that 40–70% of transplant patients taking immunosuppressive medications will develop non-melanoma skin cancer [1,2]. Skin cancers are immunogenic due to the presence of tumor-associated antigens, mutations, and/or expression of viral genes [2]. The role of the immune system in skin cancer has been well-recognized in both melanoma and non-melanoma skin cancers. This Special Issue expands on the current knowledge concerning the immunomodulation of melanoma/non-melanoma skin cancer by interferons, novel chemical compounds, and phytochemicals. It further highlights the role of immune cells and stromal cells in the tumor microenvironment and their role in the efficacy of immunotherapy. Finally, the roles of newer treatment modalities that can potentially enhance the efficacy of immunotherapy for the management of melanoma and non-melanoma skin cancers have been reviewed.

Ultraviolet (UV) radiation, primarily within the UVB range (280–320 nm), is immunosuppressive. In humans, UV-induced immune suppression is genetically determined, and those individuals in whom UV radiation does suppress cell-mediated immunity have an increased risk of developing skin cancers. Several specific immunomodulators have been identified after treatment with ultraviolet radiation and immune suppressants. They play an important role in the development, progression, and destruction of skin cancers [2]. The immunoprevention of skin cancer can be achieved by promoting antitumor immune surveillance to block the development and progression of tumors.

Ultraviolet-B-radiation-induced DNA damage, in the form of cyclobutene–pyrimidine dimers (CPD), if not repaired, cause mutations in tumor suppressor genes, resulting in the initiation of skin cancers. UVB-induced DNA damage is not only of importance in skin cancer but also plays a critical role in UVB-induced immune suppression [3]. Type I interferons (IFNs) are cytokines that play a role in the regulation of proliferation, differentiation, and immune function. Many human cancers, including skin cancer, have shown the low expression of type I IFNs and their related proteins [4,5]. Sherwani et al. have shown that type I IFNs (IFN-α/β) are involved in the repair of photodamage and the prevention of UVB-induced immune suppression. Treatment with a type I IFN agonist, imiquimod, was able to reverse immune suppression through a DNA repair mechanism [6].

The role of the innate immune system is important in the development of melanoma. There are several factors responsible for the etiology and pathogenesis of melanoma. In their review, Strashilov and Yordanov discuss the etiological factors related to melanoma. They range from exposure to UV radiation, skin phototype, pigmented nevi, the use of pesticides, geographical location, the role of genetic factors, and the state of immune suppression. They also discuss various pathways and factors that are responsible for the pathogenesis of melanoma [7]. Macrophages play an important role in this process. M1 macrophages enhance immunity and phagocytosis by targeting tumors. Moreover, M1 macrophages suppress cancer progression and metastasis [8]. Cancer cells express CD47 to avoid recognition by macrophages [9]. The organogermanium compound poly-*trans*-[(2-(carboxyethyl)germasesquioxane] (Ge-132; repagermanium) and its hydrolysate 3-(trihydroxygermyl)propanoic acid (THGP) has been reported to exhibit antitumor effects on both mice and rats in vivo [10]. In this context, Azumi et al. have shown that THGP promotes the M1 polarization of macrophages and suppresses the expression of signal-regulatory protein alpha (SIRP-α) in macrophages and CD47 in cancers. Based on these results, THGP may be considered a new regulatory reagent that suppresses tumor immunity [11]. Several studies have shown the efficacy of phytochemicals via immunostimulation involved in both innate and adaptive immune responses. Most of the studies concerning phytochemicals have been performed in in vitro systems or using nude mice, which do not truly recapitulate the involvement of the immune system. Very few studies have been performed using mouse models. In their review, Tabolucci et al. discuss the immunomodulatory functions of various phytochemicals and herbal extracts in melanoma [12].

Of all the tumors, skin cancers are extremely sensitive to immunotherapy. It can be used to treat both melanoma and non-melanoma skin cancers. Immune checkpoint inhibitors have been very effective in melanoma [2]. Another immune modulatory agent, imiquimod, has proved to be effective against primary and metastatic skin tumors, including BCC [2]. Despite the rise in the incidence of skin cancer, immunotherapy brings significant promise. Immune checkpoint inhibitor (ICI) therapies such as programmed death ligand (PDL)-1 and cytotoxic T-lymphocyte-associated protein (CTLA)-4 have been effective not only for melanoma but also for non-melanoma skin cancers. Cancer stromal cells such as tumor-associated macrophages (TAMs) and cancer-associated fibroblasts (CAFs) serve as predictors for the efficacy of ICIs. TAM-targeting therapies can hold promise for advanced melanoma and non-melanoma skin cancer [13]. Immunotherapy with ICIs has proven to be very effective for melanoma. However, it does not work for everyone. There is a need for the development of newer and more effective therapeutic modalities. Significant progress has been made in the integration of cancer immunotherapy with modular nanotechnology platforms to enhance its therapeutic efficacy and diagnostics. Current investigations using nanomaterial-mediated targeting of non-melanoma and melanoma cancers are directed at augmenting drug delivery and immunomodulation of skin cancers to induce a robust anticancer response and minimize toxic effects. The use of theranostic nanomaterials that can modulate immune mechanisms toward protective, therapeutic, or diagnostic approaches for skin cancers, along with the recent breakthroughs in nanomaterial-based immunotherapeutic modulation of skin cancer types and diagnostic potentials in personalized immunotherapies have been discussed [14]. Tamura et al. discuss their efforts in developing novel chemo–thermos–immunotherapy (CTI therapy) by conjugating a melanogenesis substrate, *N*-propionyl cysteaminylphenol (NPrCAP: amine analog of tyrosine), with magnetite nanoparticles (MNP). Their approach is based on the combination of direct killing of melanoma cells through the chemotherapeutic and thermo-therapeutic effects of NPrCAP/MNPs with exposure to an alternating magnetic field (AMF), and indirect killing through immune reaction (in situ vaccination immunotherapy). This approach also provides a mechanism for producing a tumor-specific drug delivery system (DDS) that achieves selective melanoma cell death. They plan to further enhance the immune response to CTI therapy by combining it with ICIs [15].
